# Numerical modeling in arterial hemodynamics incorporating fluid-structure interaction and microcirculation

**DOI:** 10.1186/s12976-021-00136-z

**Published:** 2021-01-19

**Authors:** Fan He, Lu Hua, Tingting Guo

**Affiliations:** 1 Department of Mechanics, School of Science, Beijing University of Civil Engineering and Architecture, Beijing, 100044 China; 2 Thrombosis Center, National Clinical Research Center for Cardiovascular Diseases, State Key Laboratory of Cardiovascular Disease, Fuwai Hospital, National Center for Cardiovascular Diseases, Chinese Academy of Medical Sciences and Peking Union Medical College, Beijing, 100037 China

**Keywords:** Fluid-structure interaction, Microcirculation, Hemodynamics, Outlet boundary condition, Numerical modeling

## Abstract

**Background:**

The effects of arterial wall compliance on blood flow have been revealed using fluid-structure interaction in last decades. However, microcirculation is not considered in previous researches. In fact, microcirculation plays a key role in regulating blood flow. Therefore, it is very necessary to involve microcirculation in arterial hemodynamics.

**Objective:**

The main purpose of the present study is to investigate how wall compliance affects the flow characteristics and to establish the comparisons of these flow variables with rigid wall when microcirculation is considered.

**Methods:**

We present numerical modeling in arterial hemodynamics incorporating fluid-structure interaction and microcirculation. A novel outlet boundary condition is employed to prescribe microcirculation in an idealised model.

**Results:**

The novel finding in this work is that wall compliance under the consideration of microcirculation leads to the increase of wall shear stress in contrast to rigid wall, contrary to the traditional result that wall compliance makes wall shear stress decrease when a constant or time dependent pressure is specified at an outlet.

**Conclusions:**

This work provides the valuable study of hemodynamics under physiological and realistic boundary conditions and proves that wall compliance may have a positive impact on wall shear stress based on this model. This methodology in this paper could be used in real model simulations.

## Introduction

As an important portion of circulatory system, microcirculation plays a key role and it should not be ignored. It serves to regulate blood flow and tissue perfusion thereby affecting blood pressure and responses to inflammation. Microcirculation presents the greatest resistance to blood flow so that the pressure wave propagation is impeded and then the reflection is produced. Thus, the fluctuation range of blood pressures gradually largens and that of flow velocity lessens from the aorta to peripheral arteries. Therefore, the pressure proximal to microcirculation is higher and the velocity is lower than those distal to microcirculation. These microvessels in microcirculation complete the change of pulsatile to steady flow by repelling the pulsations that enter from the larger arteries. It has been indicated that the cardiovascular disease risk depends on the presence and the severity of microcirculation dysfunction [1, 2]. As an important prognostic factor, microcirculation function plays a role in regulating blood flow to distal organs [3]. Without question, a single outlet boundary condition is not appropriate. However, a specific pressure or velocity profile at an outlet is often given by a traditional approach in hemodynamic simulations. Therefore, the traditional approach could not well reflect the resistance provided by microcirculation. Fortunately, we could deal with microcirculation using a porous model, which can capture the essential seepage features in microcirculation. It has been proved that a porous model could be used to exactly simulate the flow in microcirculation [4–6]. Microcirculatory effects could be addressed as outlet boundary conditions in hemodynamic simulations by a porous model, which is the likely best approach. Therefore, the function of the distal microcirculation may be represented by a seepage condition at an outlet, which can be found in our previous study incorporating microcirculation [7].

In addition, as well known, the arterial wall is compliant. The mechanical loading is contributed and a blood vessel is progressively expanded by arterial compliance [8]. Consequently, we consider arterial compliance and perform the fluid-structure interaction between blood and arterial wall. It is available for incorporating such effects that fluid-structure interaction is applied to the cardiovascular system [9]. Due to the importance of arterial compliance on flow patterns, it is significant that fluid-structure interaction in hemodynamic simulations has been pursued [10–12]. Researchers have focused on the role of arterial compliance using fluid-structure interaction and deduced that wall shear stresses are decreased by compliant wall in comparison with rigid wall [13–15]. As noted, simple boundary conditions have been employed in most previous hemodynamics modeling, such as the specification of a constant or time dependent pressure, and no perivascular support. However, physiological and realistic boundary conditions are necessary for clinical interest.

Microcirculation adjusts flow resistance, which is relevant for hemodynamic simulations. The deformation of compliant wall affects flow patterns. Given the importance of microcirculation and wall compliance on the character of the blood flow, it is remarkable that most research in vascular biomechanics has been pursued separately. The combination of arterial wall compliance and microcirculation effects on blood flow are still absent.

Arterial diseases can benefit from a numerical approach combined with fluid-structure interaction and microcirculation to determine flow alterations. In this work, numerical modeling in arterial hemodynamics is presented incorporating fluid-structure interaction and microcirculation. This manuscript aims to investigate on the effects of arterial compliance on the flow characteristics and to compare these flow variables with rigid wall when microcirculation is considered.

## The model and methodology

**Fig. 1:**
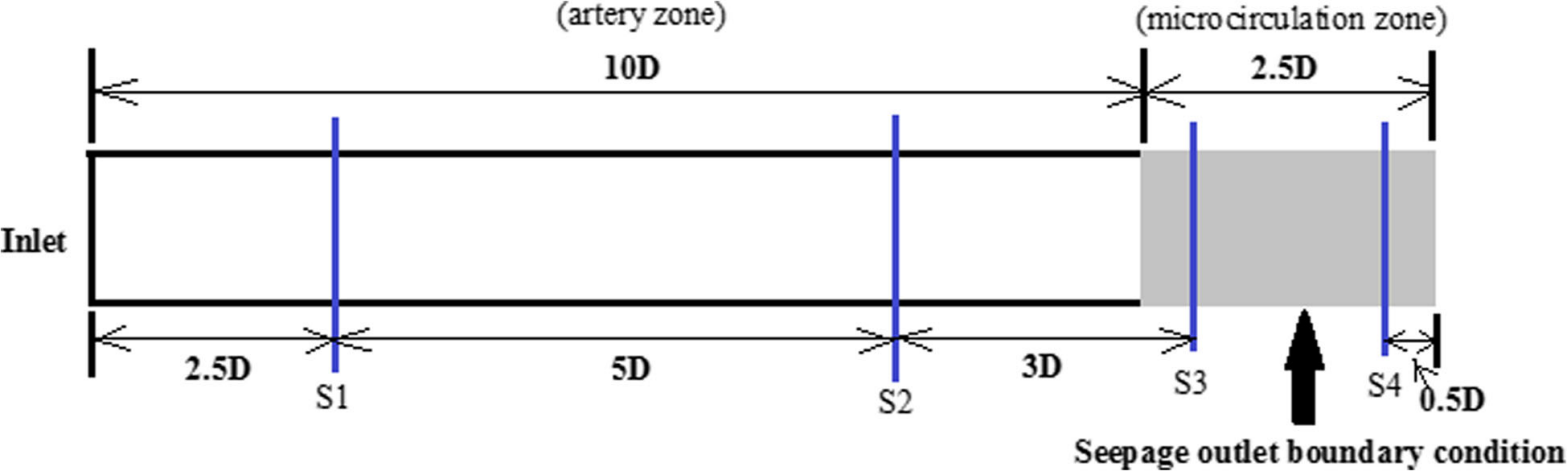
The application of a seepage condition at an outlet

Numerical modeling in arterial hemodynamics incorporating fluid-structure interaction and microcirculation is performed through constructing a standard framework in Fig. 1. The geometry is plotted to elaborate the application of a seepage condition at an outlet. In Fig. 1, D represents the diameter and equals 20 mm. The computational domains contain artery and microcirculation zones. The locations of interest sections are denoted by S1, S2, S3 and S4. Due to the arterial compliance, fluid-structure interaction is considered and the wall thickness is set to 2 mm in artery zone. However, in microcirculation zone, the rigid wall is assumed because of the tiny deformation of the vessel in microcirculation.

The Navier-Stokes equations with arbitrary Lagrangian-Eulerian formulation are used as the governing equations in the frame of artery zone [16].

1 $$ \rho \left(\frac{\partial \mathbf{u}}{\partial t}+\left(\left(\mathbf{u}-{\mathbf{u}}_m\right)\cdot \nabla \right)\mathbf{u}\right)=-\nabla p+\nabla \cdot \mathbf{T} $$

2 $$ \nabla \bullet \mathbf{u}=0 $$

Where **u** is the fluid velocity vector, **u**_*m*_ is the mesh velocity, *p* is the fluid pressure, *ρ* is the fluid density and set to 1050 kg/m^3^.

3 $$ \mathbf{T}=2\eta \left(\dot{\gamma}\right)\mathbf{D} $$

4 $$ \mathbf{D}=\frac{1}{2}\left(\nabla \mathbf{u}+\nabla {\mathbf{u}}^{\mathbf{T}}\right) $$

Where *η* and $\dot{\gamma}$ are the blood viscosity and the shear rate respectively. *η* is dependent of $\dot{\gamma}$ for a non-Newtonian fluid.

Since blood demonstrates non-Newtonian behavior, we employ the Carreau-Yasuda model in this work [17].

5 $$ \frac{\eta -{\eta}_{\infty }}{\eta_0-{\eta}_{\infty }}={\left[1+{\left(\lambda \dot{\gamma}\right)}^a\right]}^{\left(n-1\right)/a} $$

Where *η*_∞_ = 2.2 × 10^−3^ Pa·s, *η*_0_ = 22 × 10^−3^ Pa·s, *λ* = 0.110 s, *a* = 0.644, *n* = 0.392 [18].

In this simulation, a porous model is adopted to deal with microcirculation. The permeability can be computed by the following empirical equation.

6 $$ k=\frac{d^2{\phi}^3}{180{\left(1-\phi \right)}^2} $$

Where *ϕ* represents the porosity and is set to 0.5 [19]. *d* denotes microvessel’s diameter in the microcirculation and equals 8 *μm* [20].

To simplify the simulation, we make hypotheses that the arterial wall is an isotropic, incompressible and linear-elastic material. Thus, the governing equations are as follows [21].

7 $$ {\rho}_w\frac{\partial^2{d}_i}{\partial {t}^2}=\frac{\partial {\sigma}_{ij}}{\partial {x}_j}+{F}_i $$

8 $$ {\varepsilon}_{ij}=\frac{1+\nu }{E}{\sigma}_{ij}-\frac{\nu }{E}{\sigma}_{kk}{\delta}_{ij} $$

Where *σ*_*ij*_, *ε*_*ij*_, *d*_*i*_ and *F*_*i*_ are the components of the stress tensor, strain tensor, displacements and the body force acting on the solid.

In this simulation, we set the density of arterial wall *ρ*_*w*_ =1120 kg/m^3^, Young’s modulus *E* = 5 MPa [22] and Poisson ratio *ν* =0.499 [23].

The governing equations in the frame of microcirculation zone are as follows.

9 $$ \rho \left(\frac{\partial \left(\phi \mathbf{u}\right)}{\partial t}+\mathbf{u}\cdot \nabla \left(\phi \mathbf{u}\right)\right)=-\nabla \left(\phi p\right)+\nabla \cdot \left(\phi \mathbf{T}\right)-\frac{\phi^2\eta }{k}\mathbf{u} $$

10 $$ \nabla \bullet \left(\phi \mathbf{u}\right)=0 $$

**Fig. 2:**
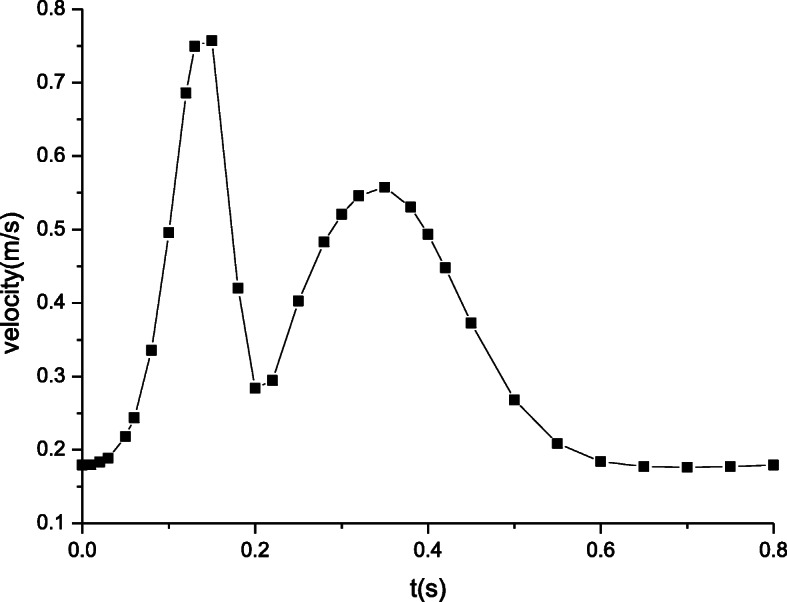
The velocity at the inlet

Figure 2 plots the velocity waveform, which is imposed at the inlet and the period is 0.8 s [22]. Subsequently, we specify the seepage microcirculation condition at the outlet. The conservative interface flux is required when blood runs from artery to microcirculation zones. Boundary conditions at the artery-microcirculation interface are given to guarantee the continuity of mass and pressure and the conservation of mass and momentum across the interface. We solve the governing equations plus associated boundary conditions for the fluid-structure interaction numerical modeling.

Both vessel ends are constrained to be entirely fixed. On the fluid-structure interface, there are the assumptions that the motions of fluid and wall are identical and there is no slipping [24].

11 $$ {\left.\left(u,v,w\right)\right|}_{\Omega}=\frac{\partial \boldsymbol{\chi}}{\partial t} $$

Where Ω denotes the wall, ***χ*** = (*r*, *θ*, *z*) are the radial, circumferential and axial position components of the deformed wall.

Two sets of commercial codes are employed to solve the fluid-structure interaction. We use ANSYS to deal with the structural problem, while the transient behavior of the fluid domain is solved by CFX. A high-resolution advection scheme is used to spatially discretize the equations. The second-order backward difference is adopted. The equations can be solved by the arbitrary Lagrangian-Eulerian algorithm, which sequentially computes fluid, structural and remeshing problems using a staggered approach. Fluid forces, solid displacements and velocities are transferred and exchanged across the fluid-structure interface through the coupling of the two sets of codes. Each time step is set to 4 × 10^− 3^ s. The variable residuals are less than 10^− 4^ and govern the number of iterations. Mesh dependency test is conducted to assure the mesh quality and simulation accuracy. We calculate the results until they become independent from the mesh. The numerical and accurate results are obtained using the proper mesh (104,400 four-node fluid elements and 13,500 eight-node solid elements). Solutions are carried out for three cardiac cycles to attain the periodic steady state. The third cycle is extracted and used for the proceeding analysis.

## Results

### Maximum pressure distributions

**Fig. 3:**
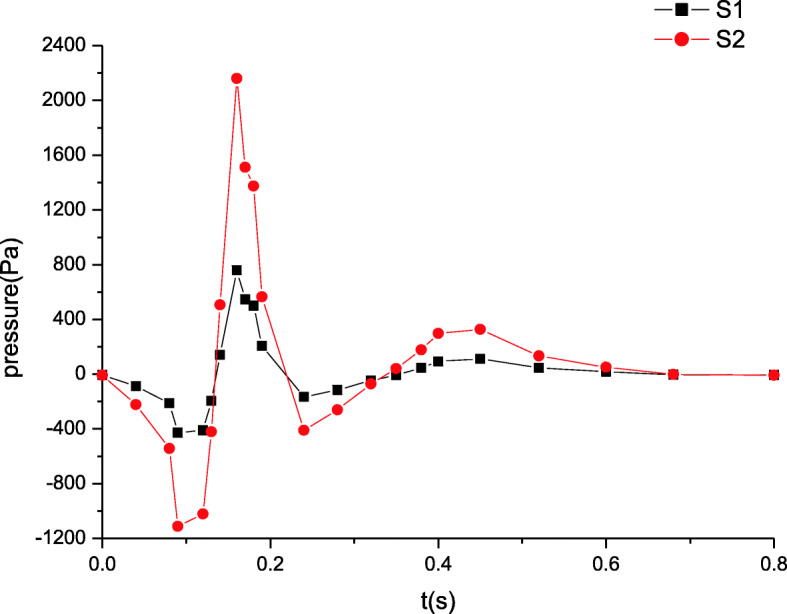
The maximum pressure distributions in artery zone

**Fig. 4:**
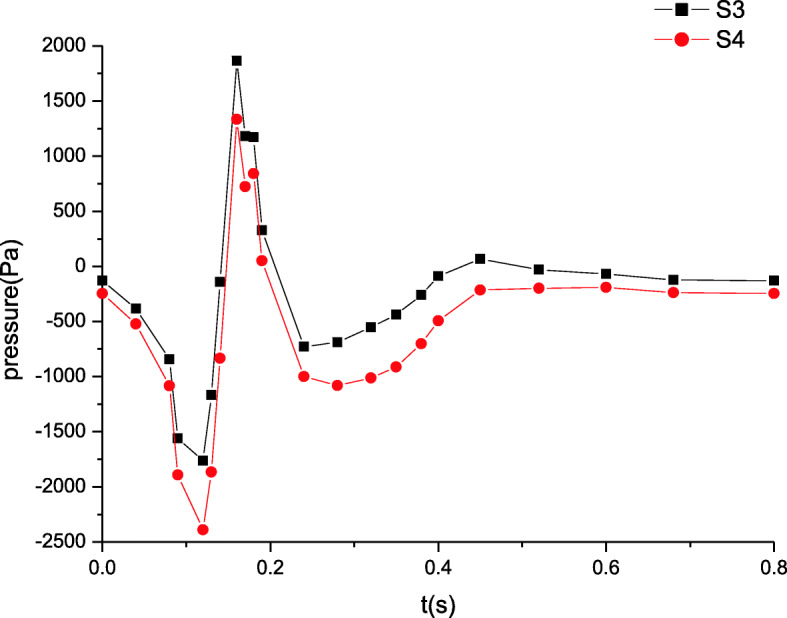
The maximum pressure distributions in microcirculation zone

The blood pressure distributions at four characteristic sections (S1, S2, S3, S4 in Fig. 1) in a cardiac cycle are depicted in Figs. 3 and 4. The comparison of the pressures at sections S1 and S2 in Fig. 3 indicates that the peak pressure increases and the bottom pressure decreases when the distance away from the inlet is increased. Thus, it is shown that the pressure amplitude progressively rises in artery zone. However at sections S3 and S4 of microcirculation zone, the pressure distributions are shown in Fig. 4. The pressures decrease gradually along microcirculation zone in Fig. 4. Clearly, it may be considered that the pressure amplitude almost maintains constant in microcirculation zone.

### Maximum velocity distributions

**Fig. 5:**
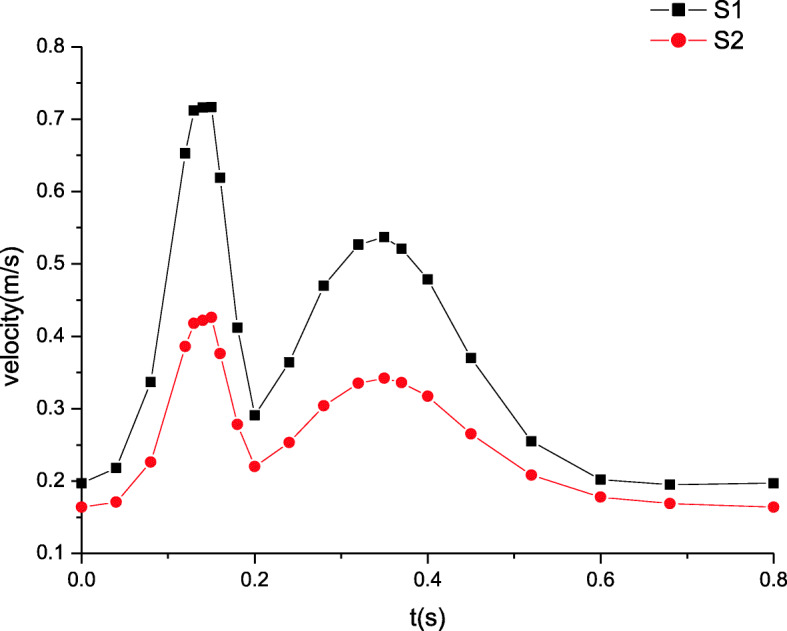
The maximum velocity distributions in artery zone

**Fig. 6:**
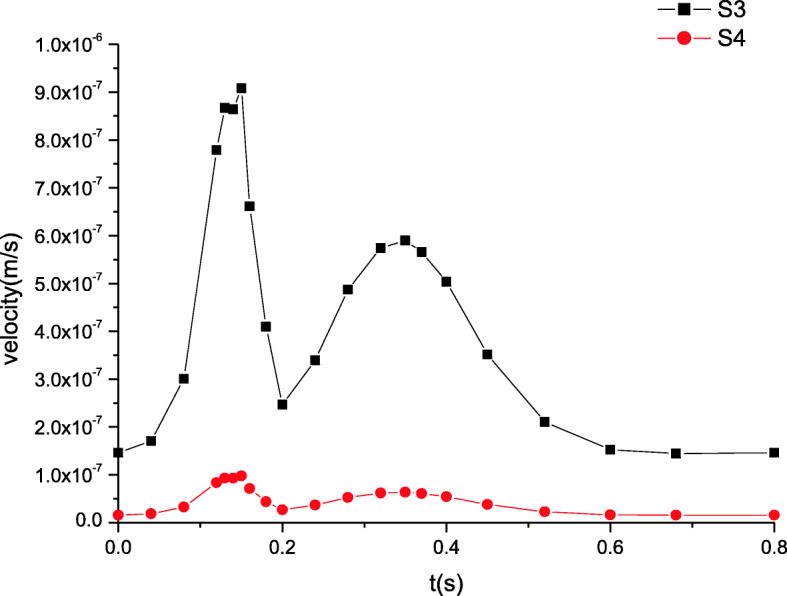
The maximum velocity distributions in microcirculation zone

The maximum velocity distributions in artery zone are plotted in Fig. 5. The inlet velocity profile determines the distributions of the time dependent velocity. In artery zone, the velocity at section S2 is lower than that at section S1. Away from the inlet, the increasing distance induces the velocity changes. The farther away the distance, the lower the velocity. Figure 6 shows the maximum velocity distributions in microcirculation zone, which resemble those in artery zone. The peak of velocity at section S1 attains to about 0.75 m/s while that at section S2 is nearly 0.45 m/s. However, the velocity in microcirculation zone is quite lower. As well known, the blood velocity in microcirculation is extremely low due to the micron scale of the microvessel’s diameter. It is seen from Fig. 6 that the velocity amplitude is also very tiny. Thus, it is often considered that there is the steady velocity in microcirculation.

### Maximum wall shear stress distributions

**Fig. 7:**
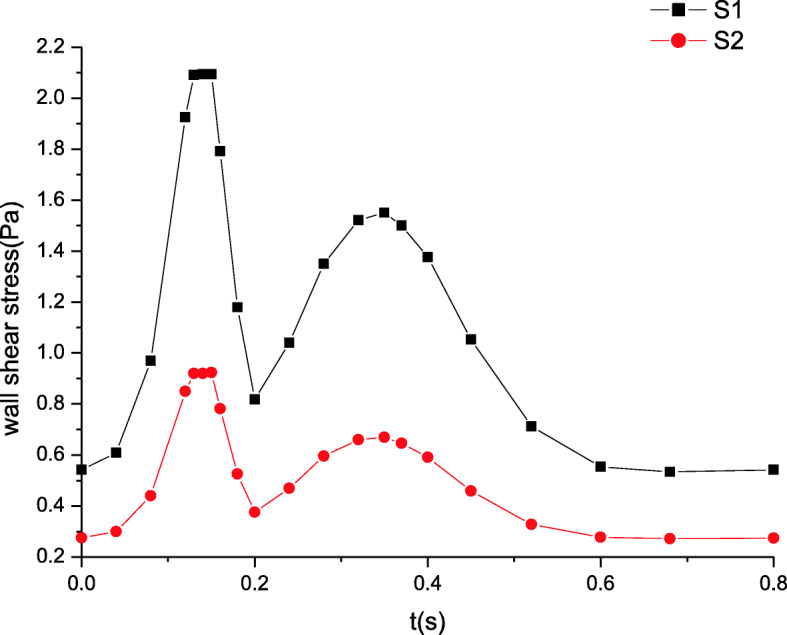
The maximum wall shear stress distributions in artery zone

**Fig. 8:**
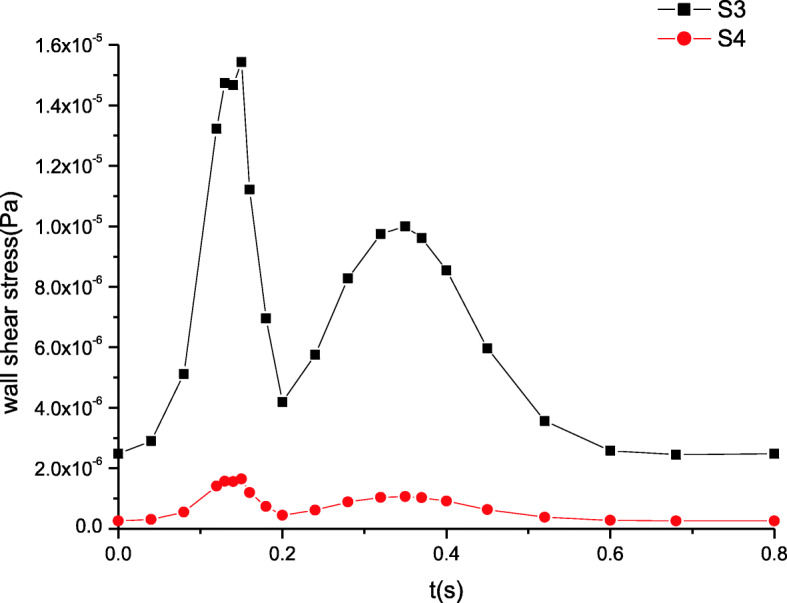
The maximum wall shear stress distributions in microcirculation zone

The maximum wall shear stress distributions are shown in Figs. 7 and 8. The wall shear stress distributions remain unchanged and the trends of their variation stay the same as those of the velocity in Figs. 5 and 6. Far from the inlet, the wall shear stress gradually decreases. The peak of wall shear stress at section S1 attains to 2.1 Pa while that at section S2 is nearly 1.0 Pa. Similarly, the wall shear stress is also deemed a constant value because the wall shear stress amplitude is tiny in Fig. 8.

We perform a sensitivity analysis to evaluate the effect of wall compliance on wall shear stress by changing Young’s modulus. The peaks of wall shear stresses at sections S1 and S2 with Young’s moduli of 4 MPa, 2.5 MPa and 1 MPa are respectively 2.2 Pa, 2.4 Pa and 2.7 Pa at section S1 while 1.05 Pa, 1.2 Pa and 1.4 Pa at section S2.

## Discussion

**Fig. 9:**
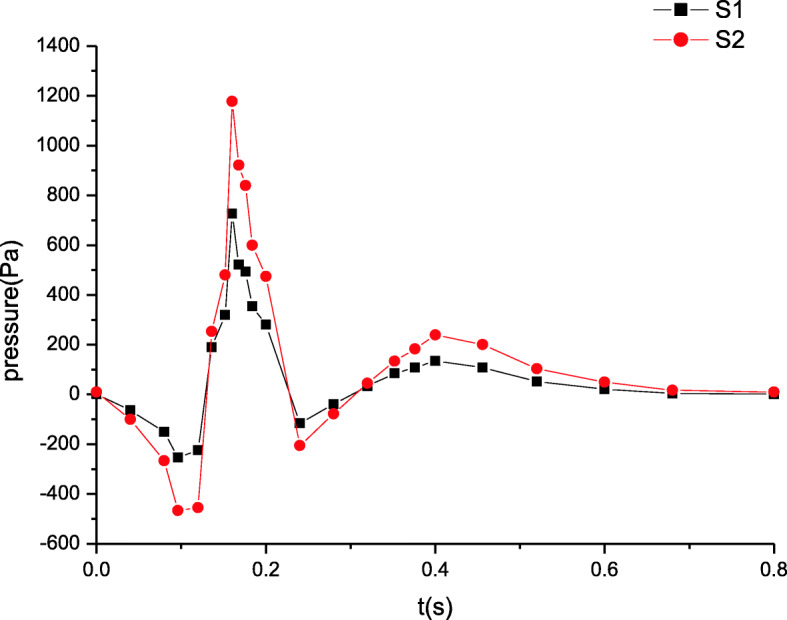
The maximum pressure distributions in artery zone under rigid wall [7]

**Fig. 10:**
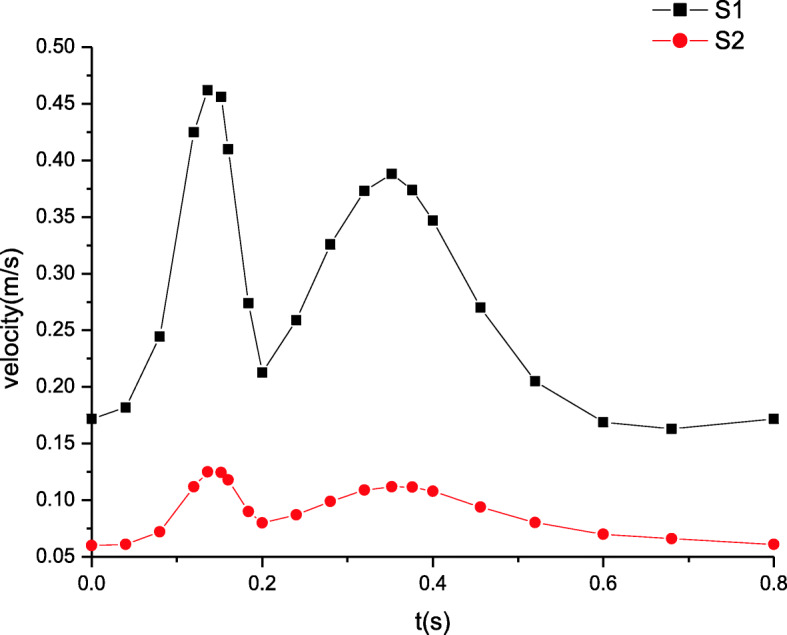
The maximum velocity distributions in artery zone under rigid wall [7]

**Fig. 11:**
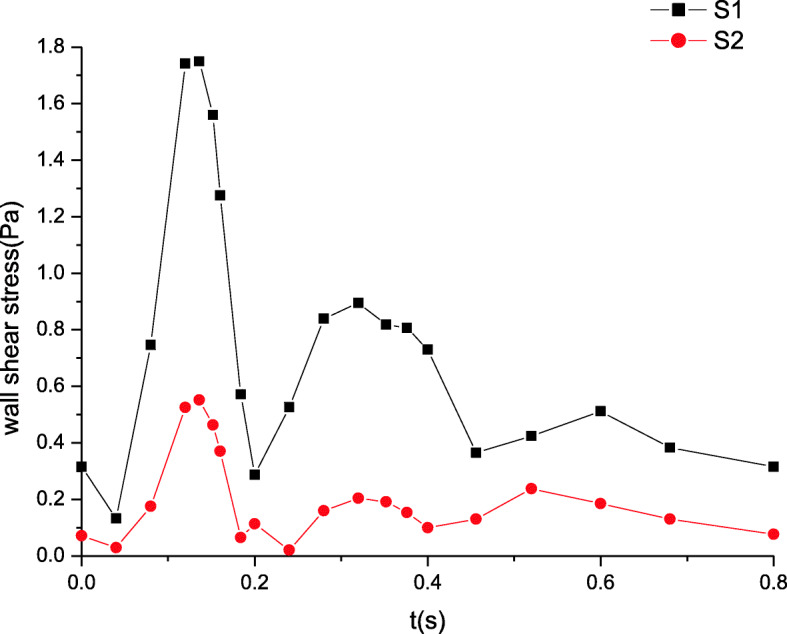
The maximum wall shear stress distributions in artery zone under rigid wall [7]

These simulated results are validated by comparing the estimated flow distributions in artery zone with Pedley’s work, which agree well with each other [22]. The results suggest that there are differences between compliant and rigid walls. The detailed results of rigid wall can be found in our previous work when a seepage condition at an outlet is considered [7]. First, the pressure amplitude under compliant wall varies more greatly than that under rigid wall. Second, the maximum pressure under compliant wall is 2200 Pa or so in Fig. 3, however that under rigid wall is only approximatively 1200 Pa in Fig. 9. Third, the peaks of velocity and wall shear stress under compliant wall are higher than those under rigid wall, which can be seen according to the comparisons of Figs. 5, 7, 10 and 11.

The most novel finding of this work is that the combination of compliant wall and seepage condition markedly enhances wall shear stress in comparison with the combination of rigid wall and seepage condition. Namely, in seepage-outlet-based hemodynamics modeling, wall shear stress can be increased by wall compliance. This result is quite different from those results obtained by a traditional approach that a constant or time dependent pressure is specified using fluid-structure interaction. The results based on the traditional approach indicate that wall shear stress can be decreased by wall compliance, which can be found in many researches [25–27]. However, it has become widely accepted that low wall shear stress contributes to atherogenesis [28, 29]. A low level of wall shear stress connects with aneurysm rupture. As an important and underlying factor, it may degenerate endothelial cells and make the aneurismal wall fragility [30, 31]. Hence, it seems that wall compliance does not have a positive impact on wall shear stress according to the traditional approach. Therefore, there will be contradiction if the traditional approach is employed in hemodynamics modeling. Fortunately, it is found that the contradiction can be solved if seepage-outlet-based hemodynamics modeling is used. This work shows that wall compliance actually makes wall shear stress increase, provided that microcirculation is prescribed at an outlet as a seepage condition. High wall shear stress can effectively avoid atherogenesis. Furthermore, it also explains that microcirculation plays an important role in regulating wall shear stress. The contribution of this finding indicates that wall compliance is advantageous to maintain health. As well known, the function of tissues is related to the result of the life optimization. It means that biological tissues adapt themselves to changes in the mechanical environment.

Although arteries exhibit viscoelastic characteristics, the assumption of linear-elasticity is made for computational convenience and it is sufficient in most physiologic and pathophysiologic cases [24]. The most well used arterial wall constitutive equation is based on linear elastic, incompressible and isotropic assumptions, which are widely recognized as an appropriate method for wall mechanics in the physiological range of intra-arterial pressures [32]. In fact, these assumptions do not change the displacement profiles of vascular wall. However, the maximum displacement using the viscoelastic model is smaller than the one with the linear elastic model with finite strain. Thus, the maximum wall shear stress is smaller for the viscoelastic model than for the linear elastic models [33]. It should be suggested that the mechanics of nonlinear and anisotropic wall is considerably required for improving the model precision. Therefore, it should be pointed out that this is a methodological paper using an idealised geometry. We will intend to apply this to patient-specific vascular geometries and thus provide some comparison with physiological curves for further validating the accuration.

## Conclusions

This investigation provides the valuable study of hemodynamics under physiological and realistic boundary conditions. The effects of wall compliance on flow patterns in hemodynamics modeling based on a seepage outlet boundary condition are detailedly discussed. Under the seepage outlet condition, wall compliance leads to the increase of wall shear stress in contrast to rigid wall, which is the novelty of this work, contrary to the traditional result that wall compliance makes wall shear stress decrease when a constant or time dependent pressure is specified at an outlet. At this point, this work proves that wall compliance positively affects wall shear stress. Certainly, it should be noted that these results are obtained based on an idealised model. The results of real model simulations should be further verified. This methodology in this paper could be used in future work.

## Data Availability

The data used to support the findings of this study are included within the article.
